# Deep Sequencing for the Detection of Virus-Like Sequences in the Brains of Patients with Multiple Sclerosis: Detection of GBV-C in Human Brain

**DOI:** 10.1371/journal.pone.0031886

**Published:** 2012-03-08

**Authors:** John D. Kriesel, Maurine R. Hobbs, Brandt B. Jones, Brett Milash, Rashed M. Nagra, Kael F. Fischer

**Affiliations:** 1 Department of Internal Medicine, Division of Infectious Diseases, University of Utah, Salt Lake City, Utah, United States of America; 2 Bioinformatics, Huntsman Cancer Institute, University of Utah, Salt Lake City, Utah, United States of America; 3 The Human Brain and Spinal Fluid Resource Center, West Los Angeles VA Medical Center, Los Angeles, California, United States of America; 4 ARUP Laboratories, Salt Lake City, Utah, United States of America; 5 Department of Pathology, University of Utah School of Medicine, Salt Lake City, Utah, United States of America; Emory University, United States of America

## Abstract

Multiple sclerosis (MS) is a demyelinating disease of unknown origin that affects the central nervous system of an estimated 400,000 Americans. GBV-C or hepatitis G is a flavivirus that is found in the serum of 1–2% of blood donors. It was originally associated with hepatitis, but is now believed to be a relatively non-pathogenic lymphotropic virus. Fifty frozen specimens from the brains of deceased persons affected by MS were obtained along with 15 normal control brain specimens. RNA was extracted and ribosomal RNAs were depleted before sequencing on the Illumina GAII. These 36 bp reads were compared with a non-redundant database derived from the 600,000+ viral sequences in GenBank organized into 4080 taxa. An individual read successfully aligned to the viral database was considered to be a “hit”. Normalized MS specimen hit rates for each viral taxon were compared to the distribution of hits in the normal controls. Seventeen MS and 11 control brain extracts were sequenced, yielding 4–10 million sequences (“reads”) each. Over-representation of sequence from at least one of 12 viral taxa was observed in 7 of the 17 MS samples. Sequences resembling other viruses previously implicated in the pathogenesis of MS were not significantly enriched in any of the diseased brain specimens. Sequences from GB virus C (GBV-C), a flavivirus not previously isolated from brain, were enriched in one of the MS samples. GBV-C in this brain specimen was confirmed by specific amplification in this single MS brain specimen, but not in the 30 other MS brain samples available. The entire 9.4 kb sequence of this GBV-C isolate is reported here. This study shows the feasibility of deep sequencing for the detection of occult viral infections in the brains of deceased persons with MS. The first isolation of GBV-C from human brain is reported here.

## Introduction

Multiple sclerosis (MS) is a chronic demyelinating disease of unknown cause, which affects the brain and spinal cord of about 400,000 individuals in the U.S. A number of viral infections of the CNS can lead to demyelination, including distemper (dogs), measles (SSPE, humans), and influenza (humans). [1] Viruses have long been suspected as causative agents in MS based on the epidemiology of the disease including geographic patterns, isolated outbreaks, and migration studies. [2], [3], [4], [5] Novel viruses that cause human disease continue to be discovered using molecular techniques including hepatitis C (1989), corona virus NL63 (2004), bocavirus (2005), and rhinovirus C group(2007). [6], [7], [8], [9] New human polyoma and arenaviruses, identified by high-throughput or “deep” sequencing of pathologic specimens, were recently discovered as causes of serious human diseases(2008). [10], [11]

To date most studies into the possible viral etiology of MS have focused on human DNA viruses. Unfortunately, because DNA viruses can become latent or quiescent within the CNS, it is difficult to discern whether latent, commensal viruses associated with MS lesions (e.g. HHV-6 and EBV) are causative or just opportunistic (i.e. “along for the ride”). To avoid the identification of latent viruses that are not transcriptionally active, the present investigation focused on viral RNA – both genomic (from RNA viruses) and message (from either RNA or DNA viruses).

Deep sequencing offers a new approach for the discovery of pathogens. In contrast to PCR, this approach allows a relatively unbiased survey of nucleic acid sequences found within a sample. Deep sequencing provides millions of short sequences less than 100 bp in length. The completion of the Human Genome Project allows subtraction of human sequences from the dataset resulting in a large number of plausibly-nonhuman sequences that can then be compared to existing databases such as GenBank. This technique was recently employed to discover novel picornaviruses linked to respiratory illness and diarrhea in children. [12], [13]

GB virus-C (GBV-C or hepatitis G virus) is a human flavivirus originally isolated from a surgeon (with the initials G.B.) who was experiencing acute hepatitis. [14] The GB viruses are approximately 10 kb in size and are most closely related to hepatitis C virus. Other flaviviruses include dengue viruses, West Nile virus, yellow fever virus, and the tick-borne encephalitis viruses. In addition to humans, GBV-A and GBV-B infect monkeys, GBV-C infects chimpanzees. The recently described GBV-D infects only bats. [15], [16] Animal inoculations suggested that GBV-C might be a cause of hepatitis, but subsequent human observations did not confirm this. GBV-C is known to be lymphotropic and has been found in human serum, muscle, lymphoid tissue, spleen, kidney and liver, but not brain (0 of 8 specimens) or spinal fluid (0 of 17 specimens). [17], [18], [19] Surveys show that active viral replication occurs in the blood in 1–2% of human blood donors while about 5% of the general population is seropositive. [20] Viral replication can persist for years at high titer (up to 50 million copies per ml blood). This virus is believed to be nonpathogenic, but has been associated with non-Hodgkin's lymphoma. [21], [22], [23] Resolution of GBV-C infection is often accompanied by antibody production against the envelope (E2) protein. GBV-C infection appears to induce antibodies that cross-neutralize HIV, probably explaining the observed survival benefit of persons infected with both viruses over those with HIV alone. [24]

We report the results of deep sequencing of diseased brain tissue (plaque) taken from deceased patients with multiple sclerosis. This method identified one occult viral infection with GBV-C in a patient who died with primary progressive MS.

## Methods

### Brain Specimens

One hundred mg pieces of cryopreserved white matter from MS plaques or control specimens were obtained from the Human Brain and Spinal Fluid Resource Center, West Los Angeles VA Medical Center and from the Rocky Mountain MS Center, University of Colorado, Denver. These specimens were collected after death and were either fresh frozen or snap frozen in liquid nitrogen. This research was submitted to the University of Utah Health Sciences IRB and, since it was performed on deidentified pathologic material, was found to be exempt from review and oversight.

### RNA Extraction and Quality Control

DNase treated RNA was extracted from the tissue sections using Qiagen RNA-lipid kits (Valencia, CA) on dry ice, under laminar airflow. Purified RNA was quantified on a Thermo Scientific Nanodrop spectrophotometer (Waltham, MA). Samples with absorbance (A_260_/A_280_) ratio <1.8 were rejected and re-extracted. Fifty ng of RNA from each sample was submitted the Huntsman Cancer Institute (HCI) Microarray Core Facility lab for analysis on an Agilent 2100 Bioanalyzer Nanochip (Agilent Technologies, USA). The extracted RNA samples were evaluated for their distribution of RNA sizes, their relative abundances, the height and relative ratio of the 28S and 18S rRNA peaks, and for RNA integrity (RIN) as determined by the Agilent software. Each sample was evaluated by an experienced technician and then independently by one of the investigators. Samples with an unfavorable electrophoresis trace or RIN were not sequenced.

### Ribosomal RNA Depletion

To enhance the detection of non-human sequences, RNA samples that passed the quality control step above were subjected to rRNA removal using the RiboMinus kit (Invitrogen Inc., Carlsbad, CA). Recovered RNA (post rRNA removal) was again quantified and subjected to Bioanalyzer analysis.

### Illumina GAII Sequencing

RNA samples that passed the RNA quality control were submitted to the Huntsman Cancer Institute Microarray Core Facility for reverse transcription, cDNA library preparation, and high-throughput sequencing. Datasets of 36 bp sequences, or reads, were obtained from each brain sample. Low complexity (LZW compressibility >0.4) reads, reads with homopolymeric stretches longer that 18 bases and those with Bustard quality scores ≤5 in more than 13 positions were excluded from the analysis. The remaining reads are defined as “high quality reads.” Reads which perfectly matched UCSC build 18 of the human genome, as judged by Bowtie alignments, were excluded from the analysis. Weaker matches to mammalian rRNA transcripts were detected and removed from the data set using MegaBLAST (word size = 12) with a database of 45S pre-rRNA and 28S sequences (gis: 1261918 and 225637499). The remaining high quality, nonhuman, nonribosomal reads were defined as “screened reads.” Comparative enrichment of viral sequence was then determined using these filtered sequences (screened reads).

### Virus-Like Sequence Filtering and Enrichment

A non-redundant database of viral sequences was built to reduce noise from highly oversampled taxa in the public databases. This nonredundant database is 98 megabases in size organized into 4080 viral taxa. All viral sequence in GenBank was obtained (more than 600,000 records). The longest sequence from each viral taxa was extracted from the input sequence set and placed in the non-redundant output set. All subsequences longer than 23 bases in the input that were compared to the sequence that was just extracted. Identities were removed from the input by replacing the identical subsequence with X's. The longest remaining sequence, thus screened, was then extracted from the input, placed in the non-redundant output and used to screen the remaining input sequences. The process was repeated until no unscreened subsequences longer than 49 bases remained in the input. The resulting database, NR_ViroBank is available online (http://fischer-lab.path.utah.edu/data/GBV-C).

MegaBLAST was used to align the brain sequences to NR_ViroBank using a low-stringency protocol. A word size of 16 was used, sequence filtering was disabled, and sequence-virus pairs with expect values ≤1.0 were considered in further analysis. The hit-rate (HR_T-S_) of a sample to a viral taxon was calculated using Equation 1 where the number of alignments to a given taxon (*A_T-S_*) is normalized by the number of filtered nonribosomal sequences obtained for the specimen (*R_S_*):
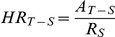
The mean and standard deviation of the normalized hit rates in the control specimens were determined for each viral taxon. The distribution of control hits was examined for normality. Taxa with non-normally distributed control hit rates were excluded from further analysis. The hit rates of each individual MS sample were then compared to the control hit-rate distribution for each taxon. Using the python programming language and scipy, the Bonferroni corrected Student's t-Test was used to quantify the statistical significance of any overrepresentations within each MS sample. Cluster and Java Treeview were used to generate an unsupervised cluster of significant sample-taxa p-value pairs. [25], [26]

### Detection of GBV-C

PCR primers were designed from 4 regions of the GBV-C genome based on the deep sequencing results. Reverse transcription was performed using independent forward and reverse gene specific priming. Primers were selected from the 5′-NTR, E2, and NS3 gene regions based on Souza et. al. with modifications as suggested by the deep sequencing results. [27] Primer sequences (5′-3′) employed were:

- 5′-NTR-1: AAGCCCCAGAAACCGACGCC, TGAAGGGCGACGTGGACCGT
- 5′-NTR-2: CGGCCAAAAGGTGGTGGATG, GTRWCGGKCTCGGTTTAACG
- E2: TGGNTCWGCCAGYTGYACCAT, DTCYCGGATCTTGGTCATGG
- NS3: TCGGCWGARYTGTCGATGCA, ACGCCGCGHACYTTTGCCCA

Quantitation of GBV-C was performed using the following (5′-NTR) primer and probe sequences:

- Forward: 5′-GGCGACCGGCCAAAA-3′ (nt 96–110)
- Reverse: 5′-CTTAAGACCCACCTATAGTGGCTACC-3′ (nt 163–188)
- Probe: 5′-FAM-TGACCGGGATTTACGACCTACCAACCCT-tamra-3′ (nt 131–158)

SuperScript® III One-Step RT-PCR System with Platinum® Taq DNA Polymerase (Invitrogen, Carlsbad, CA) was used with running conditions: 50°C 20 min, 95°C 2 min, 40 cycles of 95°C 15 sec, 58°C one minute. A standard curve was generated using log dilutions of synthetic standard GBV-C RNA (kindly provided by the Stapleton laboratory at the University of Iowa).

### Immunohistochemistry

Three anti-GBV-C antibody preparations and controls were kindly provided by the Jack Stapleton laboratory at the University of Iowa. These included pre- and post-immune rabbit polyclonal antiserum against GBV-C E2 protein [24], mouse monoclonal anti-E2 #7067 [24], and mouse monoclonal 1C4 [28], plus control mouse IgG1 (Vector Laboratories, Burlingame, CA). Frozen MS and control brain tissues were thawed and fixed in 4% paraformaldehyde. Five micron paraffinized sections were used. Antigen retrieval was performed (Vector product H-3300). The tissues were penetrated with Triton-X and endogenous peroxidase was quenched. Sections were blocked with 5% normal goat serum (Vector product S-1000). Appropriate goat anti-rabbit or goat-anti-mouse IgG secondary antibodies (Santa Cruz Biotechnology, products sc-2004 and sc-2060) were diluted 1∶500–1∶000 as recommended by the manufacturer. Signal was developed with NovaRed (Vector product SK-4800).

### Assembly of the GBV-C Isolate Sequence

Thirty-two specific primers to the MS-6 GBV-C isolate were designed using the GAII sequences that were highly homologous to known GBV-C isolates. Overlapping amplicons were obtained using RT-PCR with freshly extracted total RNA from sample 3840. Amplicons were TOPO-cloned and sequenced using an ABI 3730 sequencer. The Sanger sequences were processed and assembled using the phred, phrap and consed programs using a known GBV-C scaffold (gi: 1666805). [29], [30] The GAII reads were added to this intermediate assembly using consed.

## Results

### Brain Specimen Characteristics

Fifty frozen MS plaque and 15 control, undiseased brain specimens were extracted. Among these, 17 MS and 11 control brain specimens were selected for sequencing based on favorable RNA quality profiles. These brain specimens were collected between the years 1983 and 2004. They were kept frozen at −70 degrees until being subjected to RNA extraction in our laboratory. Characteristics of the sequenced brain specimens are shown in Table S1. The post-mortem intervals between the MS and control specimens were similar: 12.9±2.2 vs. 11.5±1.6 hours (p = 0.64 by unpaired t-testing). The MS subjects were younger than the controls at the time of specimen collection: a median of 55 vs. 69 years, (p = 0.01 by unpaired t-testing). The proportion of specimens obtained from each of the two brain banks was similar between the MS and control groups (p = 0.7 by Fisher's Exact test). The 17 sequenced MS specimens came from deceased patients that carried the diagnoses of primary progressive MS (3), chronic progressive multiple sclerosis (5), secondary progressive MS (5), relapsing-remitting MS (2), or multiple sclerosis, subtype not specified (2).

### Deep Sequencing Results

High-throughput sequencing on the Illumina GAII yielded 4–24 million 36 bp sequences (“reads”) from each of the 28 brain specimens (17 MS, 11 normal controls). These reads are designated as “high quality reads” in Table S1. These sequences represent cellular RNA, not DNA, and therefore correspond either to viral genomes or to actively transcribed genes, both human and nonhuman, present in the specimen. Human and ribosomal reads were subtracted from the dataset, providing 0.9–7.4 million screened reads for analysis. There were no significant differences in the number of high quality reads or screened reads between the MS and control specimens (p>0.5 by Mann-Whitney testing).

### Identification of Viral Candidates for Further Analysis

**Figure 1:**
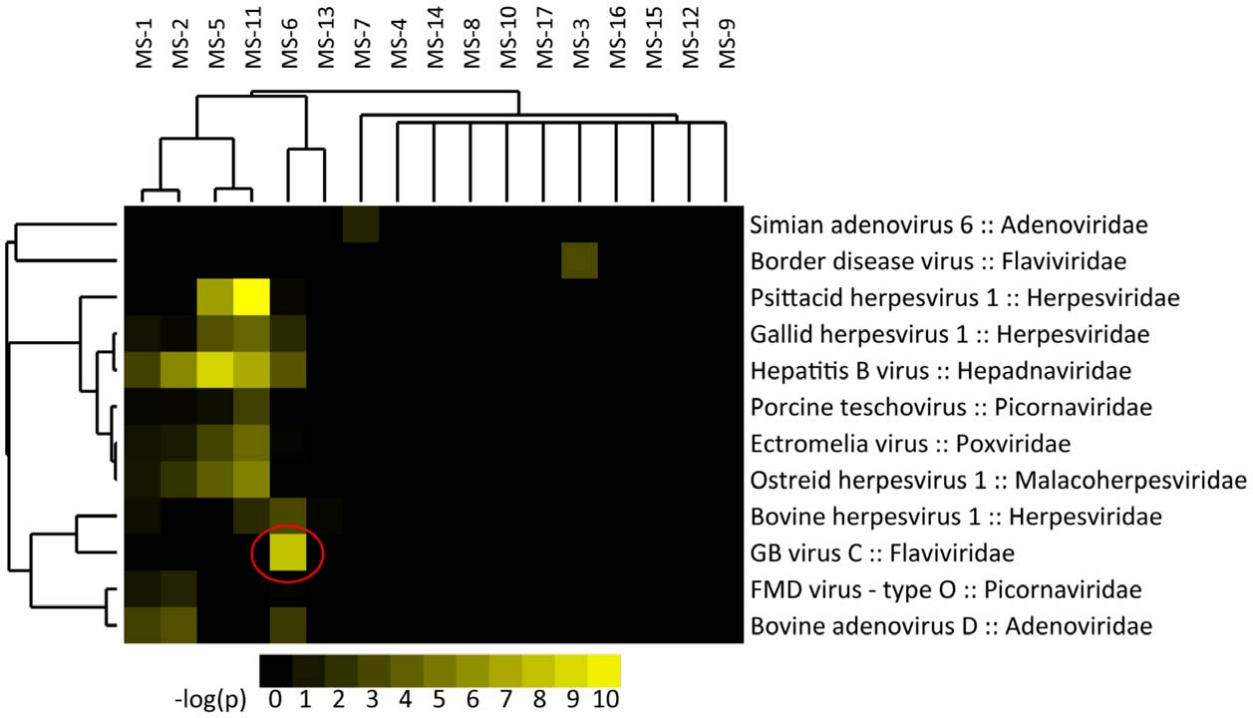
Representational Analysis. Virus-like sequences overrepresented in MS brain specimens compared with controls are displayed. Bonferroni corrected p-values beginning with 0.05 are displayed. Each row (labeled, N = 64) represents an overrepresented viral taxon. Each column (N = 17) represents a different MS brain specimen. The shaded yellow boxes represent significant hits. The GBV-C sequences confirmed by PCR in one of the specimens is circled in red.

Viral alignments were performed on the sequences derived from each of the MS and control brain specimens. Hit rates were calculated for each taxon represented in the nonredundant viral database (N = 4080). The mean and standard deviation of the hit rates in the control specimens were determined for each taxon. Only 109 out of 7947 (1.4%) viral taxa showed a non-normal distribution of control hit rates. These were removed from further consideration. Bacteriophage and plant virus taxa were excluded from further analysis due to less biologic significance compared with human and animal viruses. Retroviruses were reserved for later analysis due to the ubiquitous expression of human endogenous retroviruses (HERVS) in human specimens. Hits were observed to 2153 remaining taxa. The hit rates to each taxon for each MS sample were then compared to the control hit rate distribution for that taxon. Student's t-Test corrected for multiple comparisons using the Bonferroni method was used to quantify the statistical significance of any apparent over-representation observed in the MS samples. The 12 taxa with a least one MS sample with a over-representation more significant than p = 0.05 were placed into a hierarchical cluster (Figure 1). Over-representation in at least one taxon was observed in 7 of the 17 MS samples.

### Candidate Follow Up and Viral Sequence Recovery From Specimens

The 36 bp reads from the Illumina sequencing are not likely to identify any viruses unambiguously. Overrepresented viral taxa (shown in Figure 1) had all the deep sequencing reads from the implicated sample aligned to a database containing all GenBank sequences for the taxon of interest. The reads were then joined into contiguous regions (contigs) using SSAKE, a short read assembler. [31] Contigs generated by SSAKE were then aligned back to the taxon specific database. These newly assembled contigs were then aligned to both host and taxon specific viral databases again. Contigs that had improved alignment quality (compared to single reads) to the candidate viral sequences and worse alignment to human sequences were used to design primers for virus specific RT-PCR. The results of this analysis are shown in Table 1. This strategy identified GBV-C as a promising candidate for PCR follow up in a single MS brain sample.

**Table 1:** Virus-like sequence short read assembly.

Viral Taxon*Viral Family*	p-value^1^	Longest Aligned Contig^2^	# MS Brain Specimens^3^	Result
Simian adenovirus-6*Adenoviridae*	.04	<36 bp	1	not confirmed as viral sequence^4^
Border disease virus*Flaviviridae*	9×10^−4^	<36 bp	1	not confirmed as viral sequence^4^
Psittacid herpesvirus 1*Herpesviridae*	6×10^−12^	<36 bp	2	not confirmed as viral sequence^4^
Gallid herpesvirus 1*Herpesviridae*	6×10^−5^	<36 bp	3	not confirmed as viral sequence^4^
Hepatitis B virus*Hepadnaviridae*	2×10^−9^	60 bp	5	human sequence^5^
Porcine teschovirus*Picornaviridae*	2×10^−3^	<36 bp	1	not confirmed as viral sequence^4^
Ectromelia virus*Poxviridae*	4×10^−5^	<36 bp	3	not confirmed as viral sequence^4^
Ostreid herpesvirus 1*Maculoverpesviridae*	4×10^−6^	<36 bp	2	not confirmed as viral sequence^4^
Bovine herpesvirus 1*Herpesviridae*	9×10^−4^	<36 bp	1	not confirmed as viral sequence^4^
GBV-C*Flaviviridae*	9×10^−9^	106 bp	1	assembled, confirmed by PCR
Foot and Mouth Disease virus*Picornaviridae*	4×10^−2^	<36 bp	1	not confirmed as viral sequence^4^
Bovine adenovirus D*Adenoviridae*	5×10^−4^	<36 bp	3	not confirmed as viral sequence^4^

1 students t-test (minimum value) corrected for multiple comparisons by the Bonferroni method.

2 maximum length of all the assembled reads that aligned to the indicated virus.

3 number of MS brain specimens that had reads with significant homology (MegaBlast Expect ≤0.1) to the indicated viral sequences.

4 The assembly of short reads did not improve alignment with the specified viral sequences.

5 Assembly revealed homology to human mitochondrial and host integration sites.

### Lack of Overrepresentation of RNA from Viruses Previously Implicated in MS

Table 2 shows the analysis of deep sequencing results for viral taxa previously implicated in the pathogenesis of MS. Overall, the MS specimen hit rate was relatively high for the human herpesviruses compared to other viral taxa, probably due to the detection of RNA expressed during latency. It is also possible that these higher hit rates are driven by short sequences derived from abundant human transcripts that align to the herpesvirus taxa. However, none of the human herpesvirus nor measles virus hit rates differed significantly between the MS and control distributions.

**Table 2:** Deep sequencing results for viruses previously implicated in MS pathogenesis.

Viral Taxon^1^	MS Specimen Hit Rate Range^2^	ControlHit Rate^3^	t-Testp-value^4^
Cytomegalovirus	369–543	540±26	0.37
Human herpesvirus-6	331–447	408±8	0.062
Epstein-Barr virus	478–794	693±43	0.20
Measles virus	13.9–25.7	15.6±0. 68	*

1 Viral taxa are defined as sequences in GenBank below the level of family. This includes sequences identified as belonging to genus or species or subspecies (strain). It also includes sequences not assigned to a specific species or genus.

2 The MS Specimen Hit Rate is defined as the number of hits for a given taxon or species divided by the number of total non-ribosomal reads. This method is used to normalize these values between specimens. The range of hit rates among the 17 sequenced MS brain specimens is displayed.

3 The control specimen hit rates for each viral taxon are expressed as the mean ± SEM among the 8 control specimens.

4 Null hypothesis = the tested MS specimen falls within the expected distribution of control samples. Values provided are two-tailed. The minimum p-value among all 17 tested MS samples is shown.

* The p-value could not be calculated because the control hit rates are not normally distributed.

### Confirmation of GBV-C in One MS Brain Specimen

**Figure 2:**
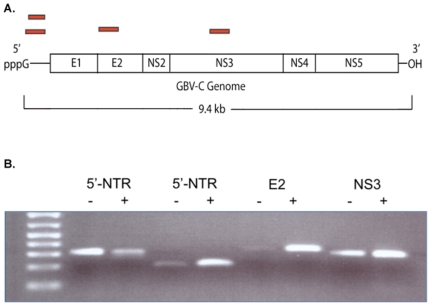
Amplification of GBV-C RNA from the brain of MS-afflicted subject MS-6. Following the failure of published primers to amplify sequences in this specimen, specific primers were constructed based on the sequences of specific reads obtained from the Illumina GAII sequencing of this specimen. [27] A. Four regions of the GBV-C genome were selected for amplification including 2 sites in the 5′ non-translated region, the E2 (envelope protein) gene, and NS3 (non-structural). B. Amplicons were derived from each of these primer sets, including + (genome) and − (replication intermediate) strands. Sequencing of 3 of these amplicons spanning 906 bp revealed identity with >100 published GBV-C isolates, indicating that this subject had a novel strain of GBV-C in her brain tissue which appeared to be replicating at the time of her death.

One subject who died with primary-progressive MS had >1000 36 bp sequences detected that mapped to GBV-C virus (hepatitis G), a human flavivirus not known to cause any persistent disease and never before detected in human brain. [14], [17], [18] This brain specimen was investigated further. An initial attempt to amplify GBV-C from this specimen using published primers was unsuccessful, later determined to be due to sequence variation of the isolate. [27] PCR primers for 3 viral regions (5′-NTR, E2, and NS3) were designed from the contigs assembled from the Illumina sequencing data on this particular subject. RT-PCR amplification of these regions revealed amplicons of the expected sizes, approximately 300 bp (Figure 2). Sequencing of the amplicons showed 96–99% identity to >100 known GBV-C sequences. Quantitative rtPCR of this brain sample showed that there were between 1.7×10^6^ and 2.4×10^6^ viral genome copies per gram of tissue. Interrogation of CSF from this subject showed 1.1×10^6^ viral genome copies/ml. Blood from this subject was not available for analysis. Anti-GBV-C antibodies were not detected in CSF from this subject (personal communication from Dr. Jack Stapleton).

### Attempts to Detect GBV-C in Other MS and Control Specimens

Specific RT-PCR amplification of 31 affected (MS) and 23 unaffected (control) brain samples was performed with each of the 3 GBV-C primer sets testing for both positive and negative strand viral RNA, using the existing GBV-C positive sample as a positive control. Both strands of GBV-C RNA were detected only in subject MS-6, both before and after ribosomal removal, and not in any of the other 53 brain RNA specimens studied. Twenty MS and seven control CSF samples were interrogated with 3 separate GBV-C specific primers (NTR-1, E2, and NS3). The MS-6 CSF sample was used as a positive control. GBV-C was amplified from none of these other 27 CSF samples.

### Full Sequence of the GBV-C Isolate

**Figure 3:**
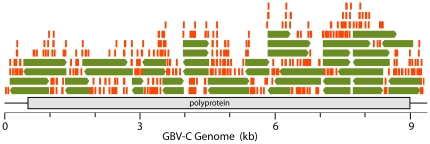
Assembly of GBV-C sequences from the deep sequencing reads. Sanger sequencing results from brain specimen MS-6 are shown in green. Deep sequencing reads from subject MS-6 that aligned perfectly (36/36 bp matches) to the GBV-C genome are displayed in orange.

The recovered sequence of GBV-C UU1 contained a open reading frame of 2842 codons, containing the structural and nonstructural genes of the virus (Figure 3). GBV-C UU1 was compared to 24 other genomic length isolates in GenBank. The isolate most similar (89.7%, nucleotide identity over the genome) to GBV-C UU1 was the East African isolate GBV-C (EA) (gi:1666805). [32] At the amino acid level the viral polyprotein was 97.7% identical to GBV-C (EA) (64 coding differences). The sequence of GBV-C UU1 has been deposited with GenBank (GB JN127373).

### Immunohistochemistry

Immunohistochemistry with controls was attempted on GBV-C infected, MS afflicted brain (MS-6); other MS brain specimens where GBV-C was not detected by PCR; and several normal brain controls. Some anti-GBV-C dependent staining of neuronal cytoplasm was observed in both infected and uninfected brain sections with the polyclonal rabbit anti-GBV-C preparation. The monoclonal Ab preparations did not specifically detect GBV-C in any of the human brain tissues examined, including that from MS-6 (shown by molecular methods to contain GBV-C RNA).

## Discussion

This work demonstrates the feasibility of deep sequencing for the detection of occult viral infections in brain tissue. This method revealed several viral candidates. The presence of one of these candidates, GBV-C, was confirmed in the brain of one of the subjects who suffered and died with progressive MS. This virus has never been detected before in human brain tissue. It's relationship with the underlying disease, primary progressive multiple sclerosis, in this single person is not clear. RNA sequences from other viruses previously implicated in the pathogenesis of MS including EBV, CMV, HHV-6, and measles, were not significantly overrepresented in the diseased brain specimens compared with controls.

GBV-C or hepatitis G virus is currently believed to be nonpathogenic. However, this virus was originally isolated from a human who was suffering from acute hepatitis. [33], [34] Prior attempts to isolate GBV-C from the brain in infected subjects were unsuccessful. [18] Other flaviviruses certainly do infect the human central nervous system including West Nile virus, dengue [35], [36], tickborne encephalitis viruses, and Japanese encephalitis virus. Even hepatitis C virus has been isolated from diseased brain in a patient with fatal demyelinating disease. [37] The epidemiology of GBV-C infection is similar to that of MS itself where infection/disease is rare in children and much more common in women of childbearing age. [38] GBV-C is, in part, sexually transmitted. [39], [40], [41] The sex predominance of GBV-C infection is about 2∶1 women∶men, again similar to MS. [42]

The presence of both positive (RNA genome) and negative (replication intermediate) strand flaviviral RNA in MS-6 suggests that the virus was actively replicating in the brain of this subject. Active replication is also supported by this subject's pathology report showing “active plaques” with a perivascular lymphocytic infiltrate (data not shown). The clinical history revealed onset of primary progressive MS beginning at age 44 in an otherwise healthy Caucasian woman. The disease was relentless causing the subject to use a wheelchair by age 55 and to become bed bound at age 60. Active replication GBV-C in the brain of MS-6 is also supported by the relatively high titer of virus observed in both tissue and CSF (1–2 million copies per ml) and the absence of anti-GBV-C antibodies in the CSF.

Actively replicating GBV-C in this subject's brain may or may not be causal. GBV-C may be found in the blood of humans for years after acute infection and it may be present at high titer – in the range of 10^8^ copies per milliliter of blood. [14], [20] Resolution of GBV-C viremia usually coincides with the development of antibodies to the virus. GBV-C replicates primarily in peripheral blood mononuclear cells, including lymphocytes, so its presence in the brain of subject MS-6 may simply reflect penetration of the damaged blood-brain barrier by serum or virus-infected lymphocytes. [18] On the other hand, persistent GBV-C infection of the brain might have manifestations not previously described, such as progressive demyelination. Animal studies demonstrate that viral infections of the CNS can result in a demyelinating disease that is similar to MS. [43], [44], [45], [46], [47] The failure to specifically detect GBV-C by immunohistochemistry in brain sections from subject MS-6 was disappointing. This may be due to a relative absence of viral antigen in these tissues or the lack of suitable reagents for the detection of GBV-C antigen in fixed, paraffinized tissue. In any case, the lack of detectable GBV-C RNA from 30 other MS brain specimens suggests that this virus will not be isolated frequently from the brains of MS patients.

Deep sequencing is a powerful tool that has revealed new pathogens. This report shows the feasibility of this technique for the study of brain tissues affected by MS. A novel isolate of GBV-C was identified here in this manner. The presence of GBV-C in this brain specimen was confirmed by designing PCR primers from the sequencing results. The deep sequencing analysis in this study was based on the early Illumina GAII technology and limited by read length (36-base pairs) and sequencing from only a single end of the library inserts. The investigators believe that further improvements in sequencing technologies, such as longer reads and perhaps paired-end strategies, will significantly simplify the bioinformatics required for future clinical viral metagenomic studies.

## Supporting Information

Table S1 **Demographic, sequencing, and disease characteristics of the sequenced MS and control brain specimens.** The source, age, sex, year of collection, post-mortem interval, and diseases associated with each specimen used for this study is shown here, along with the number of reads obtained.
(DOC)
