# Supplementary material for: Deep Sequencing for the Detection of Virus-Like Sequences in the Brains of Patients with Multiple Sclerosis: Detection of GBV-C in Human Brain
Source: PLoS One. 2012 Mar 8;7(3):e31886. doi: 10.1371/journal.pone.0031886 (PMC3297595; doi:10.1371/journal.pone.0031886)
Supplement: Table S1 — Demographic, sequencing, and disease characteristics of the sequenced MS and control brain specimens. The source, age, sex, year of collection, post-mortem interval, and diseases associated with each specimen used for this study is shown here, along with the number of reads obtained. (DOC) [file pone.0031886.s001.doc]

**Supplemental Table 1. Demographic, sequencing, and disease characteristics of the sequenced MS and control brain specimens.**

| Specimen | Source^1^ | Age^2^ | Sex | Collection Year | PMI^3^ | High QualityReads^4^ | Screened Reads^5^ | Diseases^6^ |
| --- | --- | --- | --- | --- | --- | --- | --- | --- |
| MS-1 | RM | 53 | M | 1983 | unknown | 3.8 | 0.9 | CPMS |
| MS-2 | RM | 41 | F | 2004 | 3 | 5.4 | 1.0 | RRMS |
| MS-3 | UCLA | 55 | F | 2000 | 15 | 22.1 | 5.0 | SPMS |
| MS-4 | UCLA | 76 | M | 1997 | 25 | 9.5 | 1.4 | SPMS |
| MS-5 | UCLA | 61 | M | 2000 | 10 | 5.8 | 1.2 | RRMS |
| MS-6 | UCLA | 61 | F | 2003 | 23 | 5.7 | 1.6 | PPMS |
| MS-7 | UCLA | 47 | F | 2004 | 21 | 9.8 | 0.9 | PPMS |
| MS-8 | UCLA | 64 | F | 1998 | 9 | 10.0 | 2.7 | SPMS |
| MS-9 | UCLA | 74 | F | 1999 | 20 | 24.4 | 5.6 | SPMS |
| MS-10 | UCLA | 53 | F | 2001 | 24 | 24.0 | 4.8 | SPMS |
| MS-11 | RM | 71 | F | 2003 | 4.5 | 6.3 | 1.4 | MS |
| MS-12 | RM | 43 | F | 1984 | unknown | 24.7 | 7.5 | CPMS |
| MS-13 | RM | 50 | F | 1990 | 1.5 | 7.7 | 2.6 | CPMS |
| MS-14 | RM | 30 | M | 1984 | 5 | 24.7 | 7.0 | MS |
| MS-15 | RM | 40 | F | 1987 | 3.5 | 24.8 | 6.2 | CPMS |
| MS-16 | UCLA | 70 | F | 2002 | 9 | 8.1 | 2.2 | CPMS |
| MS-17 | UCLA | 51 | F | 2001 | 20 | 9.8 | 3.0 | PPMS |
| Control-1 | RM | 57 | M | 1983 | 3.75 | 17.3 | 5.5 | Sepsis |
| Control-2^7^ | RM | 69 | F | 1987 | 8 | 5.6 | 1.5 | Neurologic Disease |
| Control-3 | UCLA | 54 | M | 2002 | 19 | 8.2 | 2.1 | CAD |
| Control-4 | RM | 69 | M | 1983 | 4.5 | 10.4 | 4.1 | Pulmonary Disease |
| Control-5 | UCLA | 76 | F | 2002 | 9 | 9.6 | 2.2 | CAD, Diabetes |
| Control-6 | UCLA | 52 | M | 2002 | 16 | 9.5 | 2.6 | Lung Cancer |
| Control-7 | UCLA | 68 | M | 2003 | 10.5 | 23.9 | 7.4 | Lung Cancer |
| Control-8 | UCLA | 73 | F | 2003 | 12 | 9.5 | 2.3 | COPD |
| Control-9 | UCLA | 76 | M | 2003 | 11 | 9.2 | 3.2 | Cardiomyopathy |
| Control-10 | UCLA | 76 | M | 2003 | 13 | 9.4 | 2.9 | Pneumonia, hydrocephalus |
| Control-11 | UCLA | 76 | M | 2003 | 20 | 9.1 | 2.9 | Stomach and Liver Cancer |

**^1^** RM = Rocky Mountain MS Bank, Denver, Colorado; UCLA = Human Brain and Spinal Fluid Resource Center, UCLA, Los Angeles, California

**^2^** Age at the time of tissue collection.

**^3^** PMI = Post-mortem interval (hours), the time elapsed between death and brain tissue collection.

**^4^** High Quality Reads = 36 bp sequences that met quality control definitions

**^5^** Screened Reads = high quality reads with human and ribosomal reads subtracted from the dataset

**^6^** Clinical and disease type information was provided by the UCLA and Rocky Mountain MS banks. CPMS = chronic progressive multiple sclerosis; RRMS = relapsing-remitting MS; SPMS = secondary progressive MS; MS = multiple sclerosis, subtype not specified; PPMS = primary progressive MS; MS = subtype not specified; CAD = coronary artery disease; COPD = chronic obstructive pulmonary disease

**^7^** This control brain specimen was excluded from the final sequence analysis due to the presence of an MS-like clinical illness, oligoclonal bands, and antinuclear antibodies.
